# REPLY BY THE AUTHORS: RE: Impact of COVID-19 on a urology residency program

**DOI:** 10.1590/S1677-5538.IBJU.2021.0060.1

**Published:** 2021-03-05

**Authors:** Alexandre Danilovic, Fabio Cesar Miranda Torricelli, Gabriel dos Anjos, Mauricio Dener Cordeiro, Marcos Giannetti Machado, Miguel Srougi, William C. Nahas

**Affiliations:** 1 Faculdade de Medicina da Universidade de São Paulo Hospital das Clínicas Departamento de Urologia São PauloSP Brasil Departamento de Urologia, Hospital das Clínicas, Faculdade de Medicina da Universidade de São Paulo, São Paulo, SP, Brasil.; 2 Faculdade de Medicina da Universidade de São Paulo Hospital das Clínicas Divisão de Urologia São PauloSP Brasil Divisão de Urologia, Hospital das Clínicas, Faculdade de Medicina da Universidade de São Paulo, São Paulo, SP, Brasil.

*To the editor,*

We thank the comments made by Dr. Atan regarding our manuscript “Impact of COVID-19 pandemic on a urology residency program” (1). Our original study demonstrated the COVID-19 pandemic negatively impacted the volume of surgeries of our urology residency program. The surgical volume decreased in minor, medium and major complex surgeries, in surgeries with less than two hours, two to four hours and more than four hours of OR time, and to all residency years, keeping the proportion of previous years. Overall reduction of surgical volume was 50.8%. The COVID-19 pandemic impacted in a similar way surgeries from all complexity and all residency years. While we were able to maintain the number of hours spent in academic activities, surgical skills training was affected by loss of around 50% of surgeries. Our residents have access to a simulation laboratory for surgical skills development, where they increased their training in laparoscopic and microscopic procedures in a dry lab and in a wet lab with porcine surgeries during pandemic (2, 3). These activities helped to maintain their technical skills. We also recommended watching high-quality surgical videos from online education library of specialty associations. Non-edited videos or semi-live surgeries are another toll used to replace live surgery. However, they are not standardized and are time consuming. We recommend non-edited videos for board certified urologist as their practice was also severely impacted by the pandemic (4, 5). Due to the absence of uniformity in urology training program, it is not possible to propose a standard protocol to mitigate the impact of COVID-19. Rather, urology residency programs should evaluate the local impact of COVID-19, considering particularities of each program, in order to fill the specific gap as soon as possible.

The Authors
